# *Achromobacter* in the Conjunctival Sac Microbiota: Potential Association With *Acanthamoeba* Keratitis Related to Orthokeratology Lenses

**DOI:** 10.1167/iovs.66.9.71

**Published:** 2025-07-30

**Authors:** Qingquan Shi, Zhenyu Wei, Jinding Pang, Ahyan Ilman Qudsi, Mingda Wei, Zijun Zhang, Yang Zhang, Zhiqun Wang, Kexin Chen, Xizhan Xu, Xinxin Lu, Qingfeng Liang

**Affiliations:** 1 Beijing Institute of Ophthalmology, Beijing Tongren Eye Center, Beijing Tongren Hospital, Capital Medical University, Beijing, China

**Keywords:** conjunctival sac microbiota, *Acanthamoeba* keratitis (AK), *Achromobacter*, orthokeratology lenses (OK)

## Abstract

**Purpose:**

*Acanthamoeba* keratitis (AK) is a severe infection linked to orthokeratology lens use, whereas the involvement of conjunctival microbiota in AK remains poorly understood. This study investigates microbiota dysbiosis in AK pathogenesis to inform microbiota-based interventions.

**Methods:**

Conjunctival swabs from 14 patients with AK and 10 healthy controls underwent 16S rRNA sequencing. Microbiome analysis compared diversity, taxa, and metabolic pathways. Functional assays quantified *Achromobacter*-enhanced *Acanthamoeba* adhesion and migration. Metagenomics and fluorescence in situ hybridization (FISH) with species-specific probes confirmed endosymbiosis.

**Results:**

Patients with AK showed reduced bacterial diversity compared with the healthy controls (*P* < 0.001) but similar richness. Relative abundance of *Achromobacter* in the AK group was higher compared to the healthy control group (*P* < 0.001). *Achromobacter* dominated microbiota among the AK group, being identified as a key biomarker via the linear discriminant analysis effect size (LEfSe). In vitro, *Achromobacter* increased *Acanthamoeba* adhesion (*P* = 0.007) and the migration area (*P* < 0.05). Metagenomic analysis and FISH further showed *Achromobacter* spp. as potential endosymbionts of *Acanthamoeba*. Kyoto Encyclopedia of Genes and Genomes (KEGG) revealed upregulated phenylalanine, fatty acid, and propanoate metabolism in the AK group (all *P* < 0.001). MetaCyc highlighted enriched pyruvate fermentation to isobutanol, aerobic respiration I, and L-isoleucine biosynthesis II in the AK group (*P* < 0.001).

**Conclusions:**

AK-associated conjunctival dysbiosis features *Achromobacter* dominance, reduced diversity, and altered metabolism. *Achromobacter* is associated with enhanced adhesion and migration of *Acanthamoeba*, indicating a possible symbiotic interaction and its potential as a biomarker and therapeutic target.

*A* *canthamoeba* keratitis (AK) is a severe ocular infection that causes intense eye pain and vision loss, significantly reducing the patient's quality of life and imposing a substantial economic burden.^1^^–^^3^ It is primarily caused by *Acanthamoeba*, a free-living protozoan commonly found in natural environments such as soil and water.^4^^,^^5^ Orthokeratology lens (OK lens) wearers are at higher risk of developing this condition due to the direct contact between the lenses and the cornea, which increases the likelihood of exposure to contaminated sources.^6^^,^^7^ With the growing popularity of contact lens use, the incidence of AK is on the rise.^8^ It is estimated that there were 12,953 cases of AK in 2023, and this number is expected to increase by 25.5% by 2073,^8^ necessitating further investigation into its pathogenesis and associated risk factors.

The critical importance of the microbiota in various ocular diseases has recently become a focus of intense research interest.^9^^–^^11^ Particularly in bacterial keratitis (BK), dysbiosis of the ocular surface microbiota has been recognized as a key pathogenic factor, with a significant decrease in *Actinobacteria* and *Corynebacteria* (known beneficial symbionts) was observed, accompanied by a notable increase in *Gammaproteobacteria*, *Pseudomonas*, *Bacteroides*, and *Escherichia-Shigella*, which were common pathogens leading to BK.^12^ However, direct evidence regarding the role of conjunctival sac microbiota in AK remains scarce. Nonetheless, as *Acanthamoeba* is an opportunistic pathogen, investigating the potential role of the microbiota in modulating its pathogenicity is a compelling avenue of research.

This study aims to systematically analyze the composition, functionality, and metabolic characteristics of the conjunctival sac microbiota in patients with AK using 16S rRNA sequencing and metagenomic techniques. By comparing microbiota profiles between healthy individuals and patients with AK, the study seeks to explore the potential relationship between microbiota dysbiosis and *Acanthamoeba* infection, as well as the associated metabolic mechanisms. These findings will not only provide insights into the pathogenesis of AK but also offer a theoretical foundation for microbiota-based prevention and therapeutic strategies.

## Methods

### Study Population

This study was conducted at the Beijing Institute of Ophthalmology, Beijing Tongren Hospital, between January 2023 and December 2024 with the approval of the Medical Ethics Committee of Beijing Tongren Hospital (TRECKY2021-024.F1), Beijing, China. In this case-control study, 14 patients diagnosed with AK by clinical manifestations and laboratory tests (positive corneal scraping or cultures for *Acanthamoeba*) and healthy controls with matched age and sex were enrolled. All participants were informed of the goals of the study, and their consents were obtained in accordance with the Declaration of Helsinki. All patients should have a history of contact lens use exceeding 1 year, and those with any medical history of infectious keratitis, ocular inflammation, trauma, or surgery within the previous 6 months, as well as those with any co-infections (bacterial, fungal, or viral), were excluded.

### Clinical Evaluation

Patient information was recorded using a standard protocol which included demographics, duration of symptoms, predisposing factors, initial diagnosis and treatments, time to AK diagnosis, clinical features, associated ocular conditions, and systemic diseases. All patients were examined by slit-lamp microscopy. Laboratory investigations for AK included corneal scrapings, optical microscopic observation after Giemsa staining, and cultures on non-nutrient agar plates overlaid with *Escherichia coli* (ATCC 25922), performed in the Department of Ocular Microbiology at the Beijing Institute of Ophthalmology. Among the 14 patients diagnosed with AK, all had positive Giemsa-stained smears of corneal scrapings, and 8 also showed positive culture results.

### Conjunctival Swabs Collection and 16S rRNA Sequencing

For patients with AK, conjunctival swabs were collected from both the affected and unaffected eyes during their initial visit, when AK was clinically suspected. The samples were cryopreserved and only processed for 16S rRNA sequencing after the diagnosis of AK was confirmed. To ensure procedural consistency, all swabs were collected by a single clinician to ensure procedural consistency. For the healthy controls, conjunctival swabs were collected only the right eyes. To prevent potential alterations in the conjunctival microbiota, no anesthetic eye drops were applied. The lower palpebral and fornix conjunctiva were swabbed three times using sterile cotton swabs (PM150, Shengtian Biotechnology, Suzhou, China), avoiding contact with the eyelid margin. The swabs were immediately placed into sterile, RNase-free cryopreservation tubes, rapidly frozen in liquid nitrogen, and then stored at –80°C for less than a month.

Following the manufacturer's protocol, bacterial genomic DNA was extracted using the E.Z.N.A. Stool DNA Kit (Omega Bio-Tek, Norcross, GA, USA), and its concentration and purity were assessed. The target region was amplified using primers 338F (5′-ACTCCTACGGGAGGCAGCAG-3′) and 806R (5′-GGACTACHVGGGTWTCTAAT-3′),^13^ specifically targeting the V3 to V4 hypervariable regions. To optimize conditions, a preliminary experiment determined the minimum cycle number required for suitable amplification, ensuring consistent and low-cycle PCR across all samples. The formal PCR reactions were conducted in a 20 µL system using Pro Taq, consisting of 10 µL 2 × Pro Taq, 0.8 µL each of Forward and Reverse Primers (5 µM), 10 ng/µL template DNA, and ddH_2_O to 20 µL. Amplification was performed on an ABI GeneAmp 9700 system (Thermo Fisher Scientific) under the following conditions: 95°C for 3 minutes; 27 cycles of 95°C for 30 seconds, 55°C for 30 seconds, and 72°C for 30 seconds; followed by 72°C for 10 minutes and storage at 4°C. Amplification products were analyzed on 2% agarose gel electrophoresis to verify band quality.

The NEXTFLEX Rapid DNA-Seq Kit was used to construct libraries from purified PCR products: (1) adapter ligation; (2) magnetic bead selection to remove self-ligated adapter fragments; (3) library template enrichment via PCR amplification; and (4) magnetic bead recovery of PCR products to obtain the final library. The sequencing was performed on the Illumina NextSeq 2000 platform (Shanghai Majorbio Bio-Pharm Technology Co., Ltd.).

After demultiplexing, the resulting sequences were quality filtered with “fastp” (0.19.6) and merged with FLASH (version 1.2.11).^14^ Quality-controlled and optimized sequences were denoised using the Divisive Amplicon Denoising Algorithm 2 (DADA2) plugin in Qiime2 under default parameters, generating amplicon sequence variants (ASVs).^15^ Sequences annotated as chloroplasts or mitochondria were removed. To minimize the influence of sequencing depth on alpha and beta diversity analyses, all samples were rarefied to 12,373 sequences per sample. Taxonomic classification of ASVs was performed using the Naive Bayes classifier in Qiime2, based on the Silva 16S rRNA gene database (version 138).^16^ Functional prediction analysis of 16S data was conducted using Phylogenetic Investigation of Communities by Reconstruction of Unobserved States 2 (PICRUSt2 version 2.2.0).^17^ The data were analyzed using the online platform of Majorbio Cloud Platform (Shanghai Majorbio Bio-pharm Technology Co., Ltd, www.majorbio.com).^18^

### *Acanthamoeba* and *Achromobacter* Culture

Peptone-yeast-glucose (PYG) medium for *Acanthamoeba* culture was prepared with 3 g peptone, 3 g yeast extract, and 6 g glucose dissolved in 400 mL distilled water. For PAS medium, ionic components (120 mg NaCl, 4 mg MgSO₄·7H₂O, 4 mg CaCl₂·2H₂O, 142 mg Na₂HPO₄, and 136 mg KH₂PO₄) were dissolved in 1000 mL distilled water. For migration assays, 1.5 g agar powder was added to 100 mL PAS medium, heated until dissolved, and then autoclaved (121°C, 15 minutes). The molten agar was poured into sterile plates at 60°C, solidified at room temperature, and stored at 4°C.

Clinical *Acanthamoeba* strains isolated from patients with AK were axenically cultured in PYG medium (30°C, log phase). Trophozoites were harvested via centrifugation (1500 rpm, 5 minutes) and PBS-washed for downstream assays. *Achromobacter xylosoxidans* (MINGZHOUBIO, BMZ012470) was isolated from bamboo slips in Changsha, Hunan Province, China. The isolate was maintained on blood agar for subsequent experiments.

### *Acanthamoeba* Genotyping

This genotyping approach was conducted with reference to a previously published study.^5^ DNA was extracted from cultured trophozoites or cysts using the FastPure Microbiome DNA Isolation Kit (DC502-01; Vazyme, Nanjing, China). The DF3 region of the 18S rRNA gene was amplified using primers JDP1 (5ʹ-GGCCCAGATCGTTTACCGTGAA) and JDP2 (5ʹ-TCTCACAAGCTGCTAGGGGAGTCA) in a 20 µL PCR mixture containing 10 µL of 2 × Flash Hot Start MasterMix (CW3007, CWBIO, Beijing, China), 1 µL of each primer, 2 µL of DNA template, and 6 µL of double-distilled water. The PCR cycling conditions were: initial denaturation at 95°C for 5 minutes, followed by 35 cycles of 95°C for 60 seconds, 62°C for 45 seconds, and 72°C for 45 seconds, with a final extension at 72°C for 5 minutes. PCR products were verified by 1.5% agarose gel electrophoresis and subjected to Sanger sequencing. The obtained sequences were compared to reference genotypes available from The Ohio State University *Acanthamoeba* database (https://u.osu.edu/acanthamoeba/) to determine genotype. Phylogenetic analysis was performed using ClustalW alignment and the maximum likelihood method in MEGA11, and visualized using the ITOL online tool.

### Adhesion and Migration Assays

The adhesion assay was used to evaluate *Acanthamoeba* binding to human corneal epithelial (HCE) cells. HCE cells (ATCC PCS-700-010; American Type Culture Collection) were cultured in DMEM (Gibco, USA) supplemented with 10% fetal bovine serum (FBS; Gibco, USA), and 1% penicillin/streptomycin (Gibco, USA) under standard conditions (37°C, 5% CO_2_). HCE cells were seeded in 48-well plates and cultured for 24 hours to form confluent monolayers. *Acanthamoeba* trophozoites (1 × 10⁵ cells/well) were added to each well and incubated at 37°C under 5% CO₂ for 1 hour. Unbound *Acanthamoeba* were removed by gentle washing with 500 µL PBS per well. Bound *Acanthamoeba* were quantified using a hemocytometer: adhesion rate = [1 − (unbound *Acanthamoeba* / total *Acanthamoeba*)] × 100%.

To investigate the potential impact of *Achromobacter* on *Acanthamoeba* motility, we performed an in vitro migration assay. *Achromobacter xylosoxidans* was cultured and adjusted to 0.5 McFarland standard, and 200 µL of bacterial suspension was spread onto PAS plates. As a control, 200 µL of PBS was spread on separate plates. *Acanthamoeba* trophozoites (10⁴ cells) were inoculated at the center of both plates. The plates were incubated at 30°C. The migration distance was recorded at 24, 48, and 72 hours, recording the position of the farthest amoebe in at least 4 directions. These positions were used to generate a smooth migration curve, and the migration area was quantified using ImageJ (version 1.54p).

### Metagenomic Sequencing and Validation of *Acanthamoeba* Endosymbionts

Genomic DNA was extracted using the QIAamp DNA Mini Kit (QIAGEN) following standard protocols. The extracted DNA was fragmented to 350 bp using a Covaris ultrasonic processor and prepared for library construction, including end repair, A-tailing, adapter ligation, PCR amplification, fragment selection, and purification. Library preparation and sequencing were performed by Novogene Bioinformatics Technology Co., Ltd. on the Illumina platform using a PE150 strategy. The raw FASTQ data were quality-checked with Fastp version 0.19.7, and potential *Escherichia coli* (ATCC 25922) sequences were removed using Kneaddata version 0.12.0. The filtered sequences were assembled using SPAdes version 3.15.5, and the quality of the assembled genome was assessed using QUAST version 5.2.0. The resulting FASTQ data were then uploaded to the Chan Zuckerberg ID (CZID), a cloud-based metagenomics platform, and analyzed using the Nanopore mNGS Pipeline version 0.7 with default parameters, including human genome filtering.^19^ The relative abundance was presented as normalized taxon reads per million (NTrPM) values. To monitor potential contamination, both negative (PBS only) and positive controls (*Achromobacter xylosoxidans* only) were included throughout the DNA extraction and library preparation processes.

Fluorescence in situ hybridization (FISH) was performed to detect *Achromobacter* endosymbiosis in *Acanthamoeba* trophozoites. The Cy3-labeled probe Ach-221 (5′-CGC TCY AAT AGT GCA AGG TC-3′; Sangon Biotech, Shanghai, China) targeting *Achromobacter* 16S rRNA was hybridized to methanol-fixed trophozoite smears.^20^ After fixation in methanol for 30 minutes, permeabilization with proteinase K (20 µg/mL, 37°C, for 5 minutes), pre-hybridization (hybridization buffer, 37°C, for 1 hour) and hybridization (50 ng/µL Cy3-labeled Ach-221 probe, 55°C, for 12 hours), slides were washed sequentially with SSC buffers (2 ×, 1 ×, and 0.5 ×) and counterstained with DAPI (1 µg/mL, for 5 minutes). Probe specificity was validated using *Achromobacter* as a positive control and *Staphylococcus aureus* isolated from a patient with keratitis as a negative control. Imaging was conducted using a fluorescence microscope equipped with Cy3 (ex/em 550/570 nm) and DAPI (358/461 nm) filter sets.

### Statistical Analysis

Alpha diversity indices (Shannon evenness, Simpson evenness, Chao1, and observed ASVs) were calculated using mothur version 1.46.1.^21^ Beta diversity was analyzed via non-metric multidimensional scaling (NMDS) based on Bray-Curtis distances, with statistical significance assessed by PERMANOVA (9999 permutations) using vegan version 2.6-4. Differential taxa were identified by linear discriminant analysis effect size (LEfSe; linear discriminant analysis [LDA] score >3, Kruskal-Wallis *P* < 0.05 after Benjamini-Hochberg correction).^22^ Phenotype prediction was performed with BugBase version 1.0.^23^ Continuous variables were tested for normality using Shapiro-Wilk test. Normally distributed data were compared with the Student’s *t*-test (two-group) or ANOVA with Tukey’s post hoc test (three-group). Non-normal data were analyzed using Wilcoxon rank-sum test (two-group) or Kruskal-Wallis test with Dunn’s post hoc correction (three-group). Categorical variables were compared via Fisher’s exact test. All *P* values were adjusted for multiple comparisons using false discovery rate (FDR). Analyses were performed in R software version 4.4.2, with boxplots displaying median (interquartile range [IQR]) and significance defined as *P* < 0.05.

## Results

### Patient Information

Conjunctival microbiome samples were obtained from 28 eyes of 14 patients with AK and 10 eyes of 10 healthy controls. Patients with AK (*n* = 14) and the healthy controls (*n* = 10) had comparable age (13.9 ± 2.4 vs. 14.4 ± 2.8 years, *P* = 0.664) and sex distribution (64.3% female patients and 60.0% female patients, *P* > 0.999).

The median onset time for AK was 30 days (range = 2–180 days). Among the patients with AK, 57.1% reported prior exposure to tap water or saline. Two patients had bilateral involvement, whereas the rest were unilateral. The median best-corrected visual acuity (BCVA) was 0.1 (range = hand motion to 0.8) for affected eyes, and 1.0 (range = 0.2 to 1.0) for unaffected eyes. The detailed clinical characteristics are provided in the Table.

**Table.** Demographics and Medical History of Patients With AK

ID	Sex	Age, y	Affected Eye	Risk Factor	Onset Time, d	Initial BCVA	Strain Number of *Acanthamoeba* (Genotypes)	Sample Number of Conjunctiva
1	F	17	OS	Tap water	15	0.1/1.0	320 (T4A)	A1, U1
2	F	16	OS	Tap water	30	0.8/1.0	316 (T4E)	A2, U2
3	M	13	OS	Saline fluid	60	HM/0.2	312 (T4B)	A3, U3
4	F	16	OS	Tap water	30	—	315 (T4A)	A4, U4
5	M	10	OS	—	20	HM/1.0	—	A5, U5
6	F	16	OU	Tap water	15	0.1/0.1	—	A6, A7
7	M	11	OS	—	90	0.5/1.0	304 (T4A)	A8, U6
8	F	17	OS	Saline fluid	30	—	—	A9, U7
9	F	15	OD	—	10	—	306 (T4A)	A10, U8
10	F	14	OD	Fever	150	—	302 (T4D)	A11, U9
11	F	12	OD	—	2	—	300 (T4B)	A12, U10
12	M	10	OU	—	30	0.1/0.2	—	A13, A14
13	F	14	OD	Tap water	30	0.8/HM	—	A15, U11
14	M	14	OD	Trauma	180	0.2/HM	—	A16, U12

BCVA, best corrected visual acuity; F, female; HM, hand move; M, male; OD, right eye; OS, left eye.

### Changes of Conjunctival Sac Microbiota in Patients With AK 

**Figure 1.**
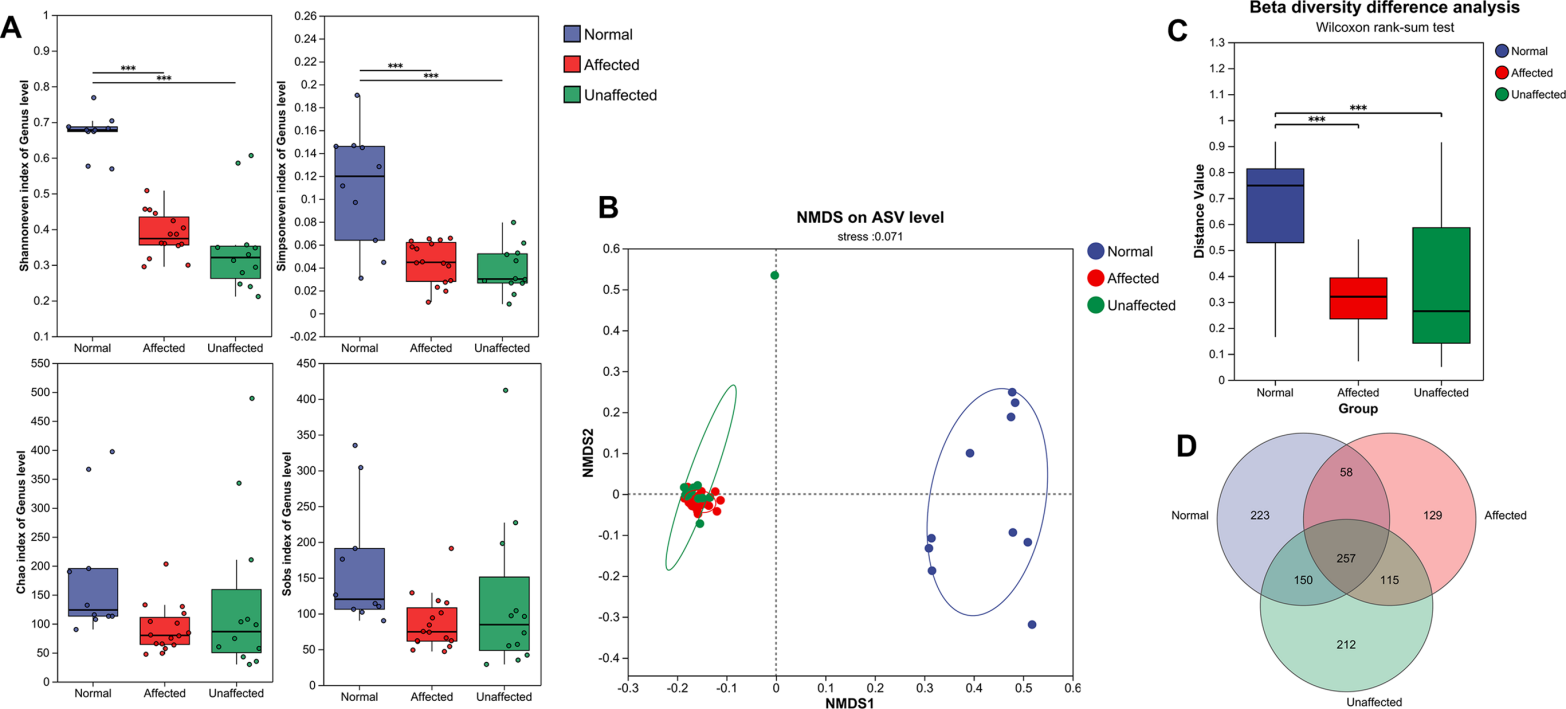
(**A**) Alpha diversity indices (Shannon's even, Simpson's even, Chao, and Sobs) across the healthy control, affected, and unaffected groups. Significant differences (*P* < 0.001) were observed among the three groups. (**B**) Non-metric multidimensional scaling (NMDS) plot illustrating clear separation and significant beta diversity differences among the three groups. (**C**) Beta diversity difference analysis among the three groups based on PERMANOVA tests, confirming the statistical significance (*P* < 0.001) of the compositional differences. (**D**) illustrating the overlap of amplicon sequence variants (ASVs) in the microbiota among the three groups, indicating the presence of both shared and unique microbial features.

Conjunctival swabs were collected and analyzed among three groups: the affected group (eyes affected by *Acanthamoeba*, *n* = 16), the unaffected group (eyes unaffected by *Acanthamoeba* in patients with AK, *n* = 12), and the healthy control group (healthy controls, *n* = 10). Sequencing generated 2,861,554 high-quality reads (75,304 per sample), yielding 11,704 ASVs annotated to 46 phyla and 1224 genera (Supplementary Table S1). As for the alpha diversity, significant differences were observed among the three groups. The Shannon's even index and Simpson's even index were statistically significantly lower in the affected and unaffected groups compared to the healthy control group (*P* < 0.001), indicating reduced bacterial diversity in both the affected and non-affected eyes of patients with AK. However, no significant differences in the Chao index and Sobs index found among the three groups suggested similar bacterial richness levels among the three groups (Fig. 1A).

As for beta diversity, NMDS revealed distinct clustering between the healthy control group and the AK-affected/unaffected groups (Fig. 1B). Although the affected and unaffected eyes showed substantial overlap with no significant difference (PERMANOVA, *P* > 0.999), both significantly differed from the healthy controls (*P* < 0.001; Fig. 1C). Therefore, the affected and unaffected groups were subsequently combined into a single AK group for microbial composition and functional analysis. To gain a clearer understanding of the shared diversity among the groups, a Venn diagram was constructed to illustrate the overlaps. The results revealed that only 187 of the total 1144 ASVs were shared across all groups, with the healthy control group exhibiting a higher number of unique ASVs (Fig. 1D).

### *Achromobacter* Dominates the Conjunctival Sac Microbiota in Patients With AK 

To investigate the microbial differences between the infection and healthy control groups, we analyzed the relative abundances at the order, family, and genus levels. At the order level, *Burkholderiales* was the most dominant order across both groups (Supplementary Fig. S1A). At the family level, *Alcaligenaceae* was the most abundant family in the AK group, whereas *Comamonadaceae* was the most dominant family in the healthy control group (Supplementary Fig. S1B).

**Figure 2.**
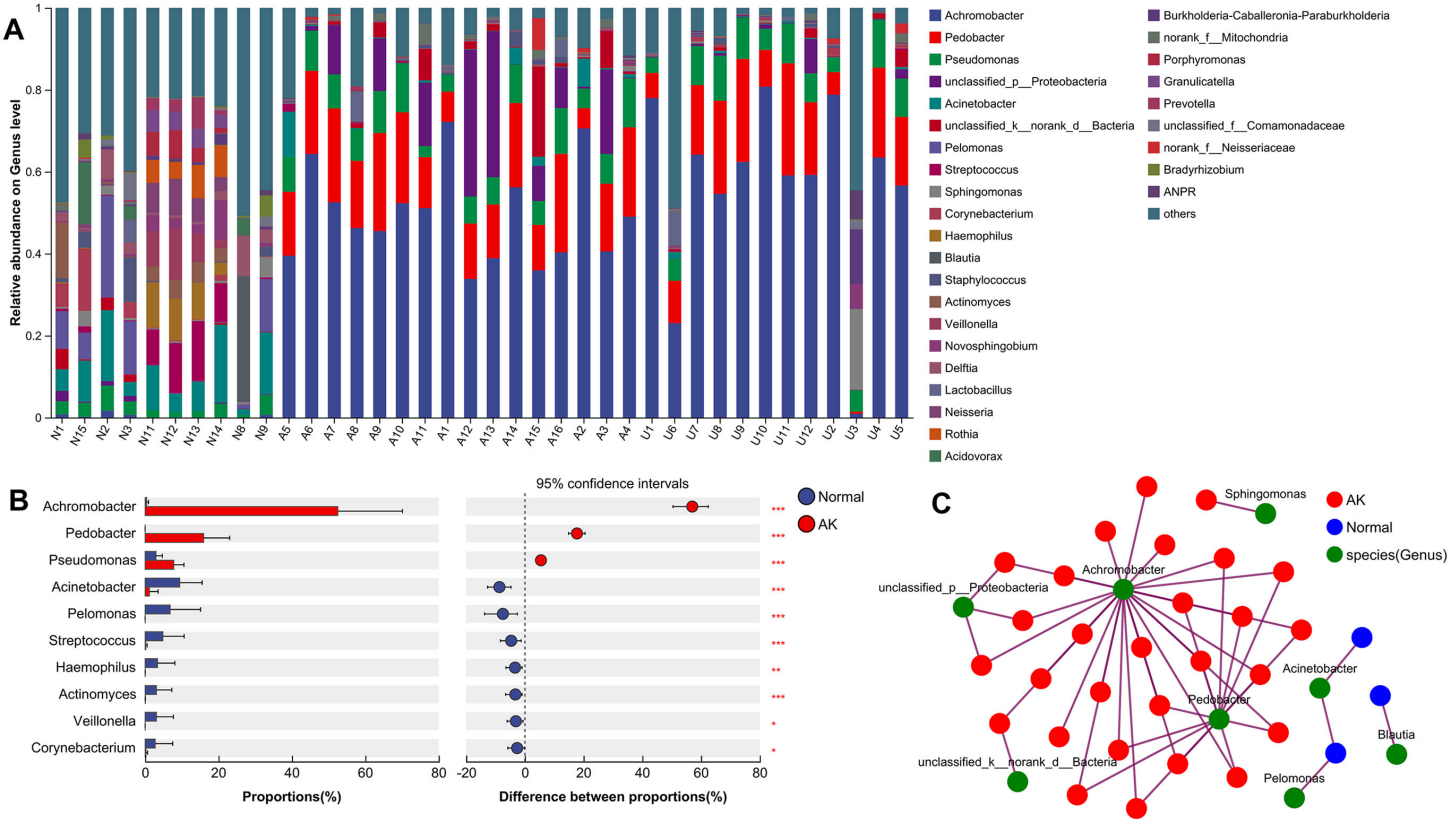
Comparative analysis of bacterial genera between the healthy control and the AK groups. (**A**) Bar plot showing the relative abundances of bacterial genera in the healthy control and the AK groups. (**B**) Statistical comparison of the proportions of key genera between the two groups. (**C**) Co-occurrence network illustrating the interactions among bacterial genera. Red = the AK group; blue = the healthy control group; and green = species across groups. Significant differences denoted as * *P* < 0.05, **P < 0.01, and ***P < 0.001.

When further investigating the genus level, we found that *Achromobacter*, *Pedobacter*, *Pseudomonas*, *Acinetobacter*, and *Pelomonas* were the five most dominant genera across the two groups (Fig. 2A). Notably, the relative abundance of *Achromobacter* was significantly higher in AK group than that in the healthy control group (median = 52.5% vs. 0.4%, *P* < 0.001; Fig. 2B). Likewise, *Pedobacter* was more abundant in the AK group compared to that in the healthy control group (median = 15.9% vs. 0%, *P* < 0.001). In contrast, *Acinetobacter* was significantly less abundant in the AK group than that in the healthy control group (median = 1.1% vs. 9.4%, *P* < 0.001). To further explore whether the increased abundance of *Achromobacter* was associated with local infection or a systemic microbial alteration, we compared the relative abundances among the affected group, the unaffected group, and the healthy control group (Supplementary Fig. S2). Interestingly, the affected and unaffected groups exhibited significantly higher *Achromobacter* levels compared with those in the healthy control group (both *P* < 0.001), with no significant difference between the affected and unaffected groups (*P* > 0.999). Additionally, the co-occurrence network analysis (Fig. 2C) revealed marked differences in bacterial interactions between the two groups. In the AK group, *Achromobacter* and *Pedobacter* emerged as central nodes, forming strong connections with other genera, suggesting their key roles in the altered microbial community. By contrast, the healthy control group exhibited a more balanced network structure, with no genera occupying dominant positions.

**Figure 3.**
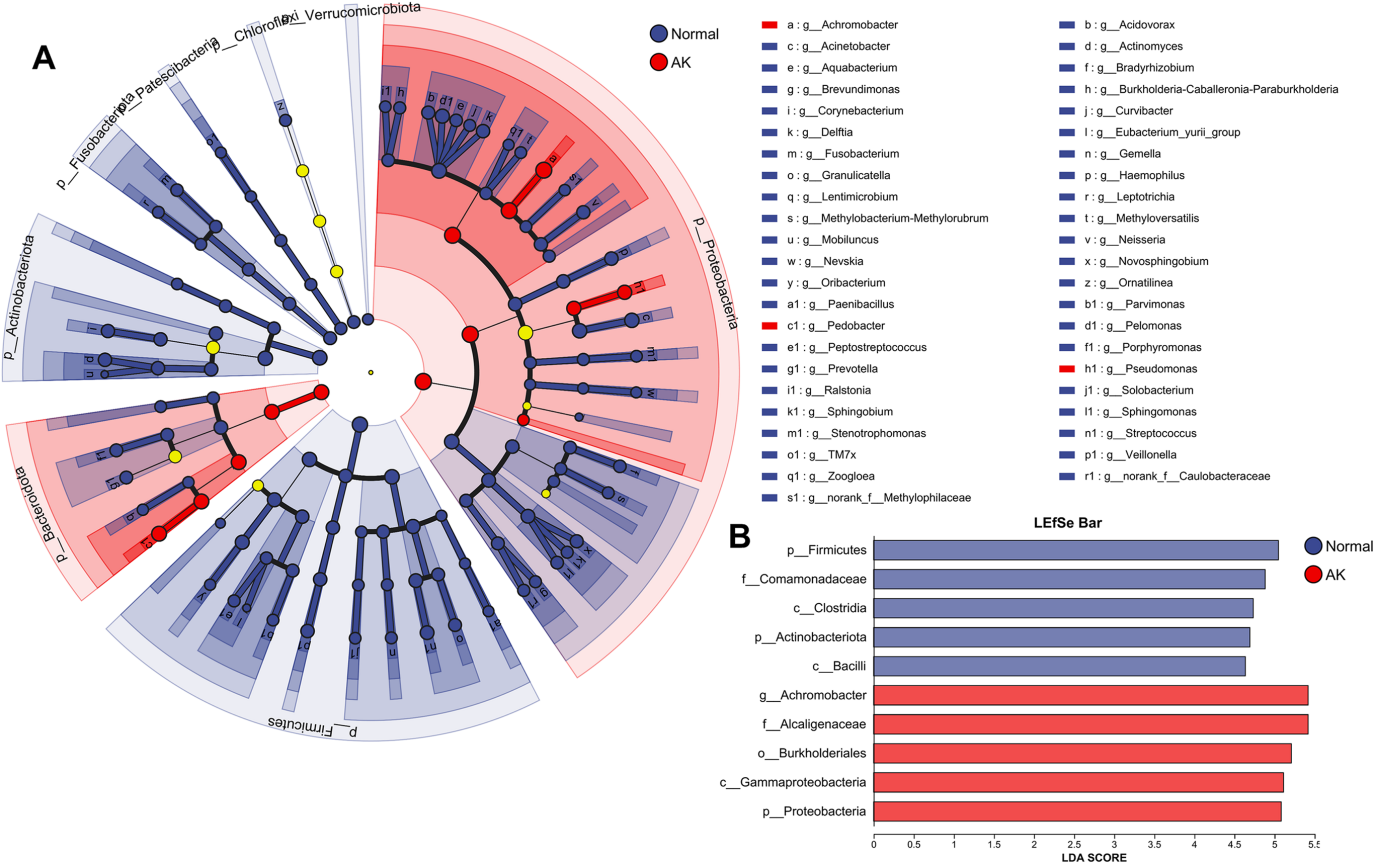
LEfSe analysis of bacterial taxa distinguishing the healthy control and the AK groups. (**A**) Cladogram shows the phylogenetic distribution of bacterial taxa with significant differences between the healthy control (*blue*) and the AK (*red*) groups. The *yellow nodes* indicate taxa with no significant difference. (**B**) LDA score bar plot displaying the effect size of significantly different taxa in the healthy control and the AK groups. The LDA score reflects the contribution of each taxon to the group separation.

The LEfSe analysis identified significant bacterial taxa distinguishing the healthy control group from the AK group at multiple taxonomic levels (Fig. 3). In the cladogram (see Fig. 3A), the phylum *Proteobacteria* was a dominant marker in the AK group, whereas *Firmicutes* were enriched in the healthy control group. At the genus level, *Achromobacter* was identified as a key marker for the AK group, showing the strongest association with this condition. The LDA score bar plot (see Fig. 3B) quantified the effect size of these taxa, with *Achromobacter*, *Burkholderiales*, and *Proteobacteria* being the most strongly enriched in the AK group, whereas *Firmicutes*, *Comamonadaceae*, and *Clostridia* were predominant in the healthy control group. These results highlight a distinct microbial community structure between the healthy control and the AK groups, with a clear shift toward *Achromobacter*-dominated communities in the AK group, suggesting their potential association with disease development or progression.

### Altered Conjunctival Microbiota Phenotypes Correlate With *Acanthamoeba* Invasion

**Figure 4.**
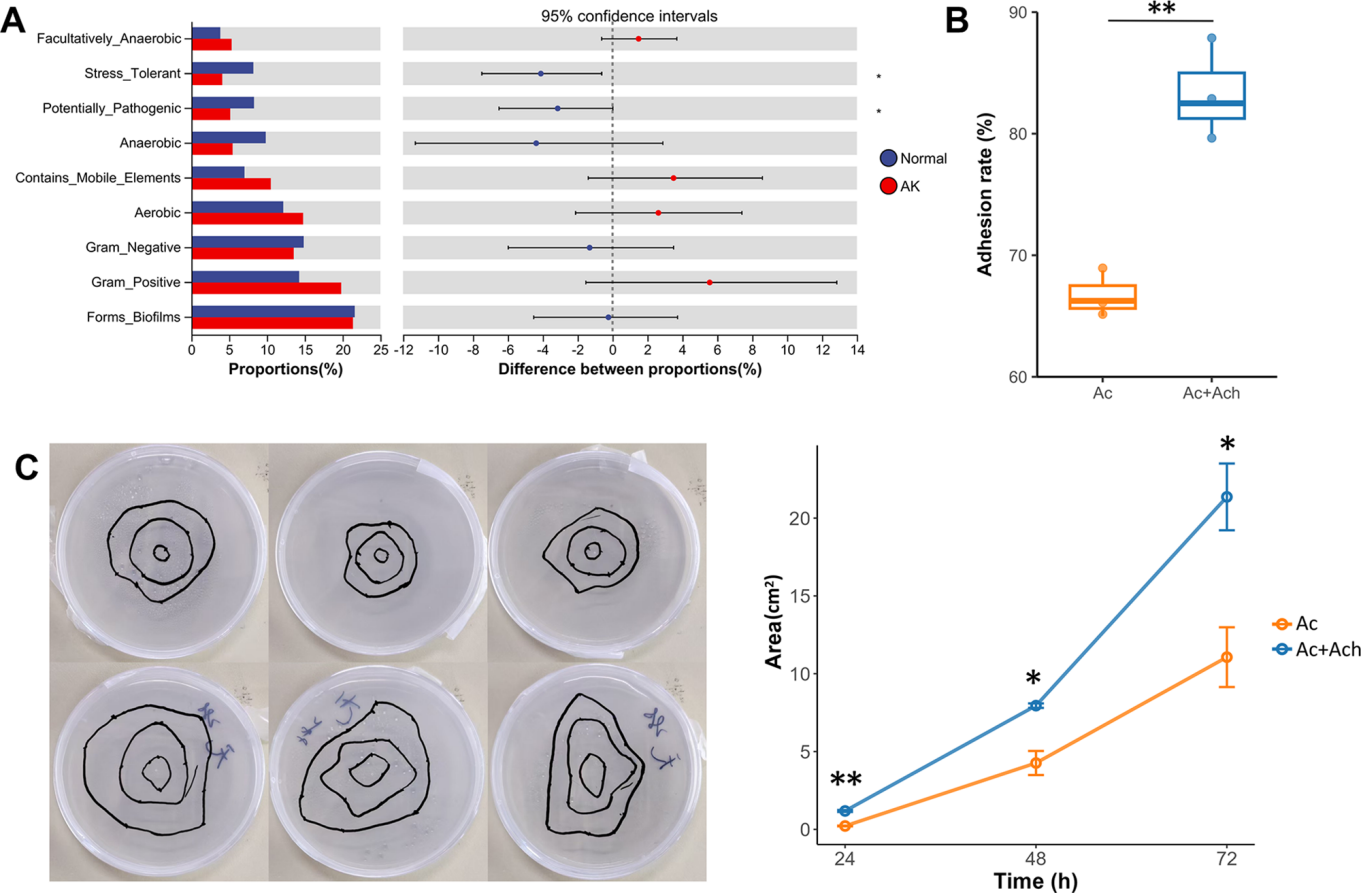
Altered conjunctival microbiota phenotypes facilitate the *Acanthamoeba* invasion. (**A**) Comparison of BugBase phenotypes between the healthy control and the AK groups. (**B**) Quantification of *Acanthamoeba* adhesion to corneal epithelial cells between the *Acanthamoeba* alone (Ac) and the *Acanthamoeba* with *Achromobacter* (Ac+Ach) groups. (**C**) *Acanthamoeba* migration assay in the Ac (*top*) and Ac+Ach (*bottom*) group. The *left panel* shows representative images of *Acanthamoeba* migration in the Ac and Ac+Ach groups at 24, 48, and 72 hours. The *right panel* quantifies the migration area (*n* = 3). * Represents 0.01 < *P* < 0.05 and ** represents 0.001 < *P* < 0.01.

Through the potential prediction of bacterial phenotypic functions in the healthy control and the AK groups, nine potential microbial phenotypes were identified, including aerobic, anaerobic, mobile element-containing, facultatively anaerobic, biofilm-forming, Gram-negative, Gram-positive, potentially pathogenic, and stress-tolerant phenotypes (Fig. 4A). Interestingly, compared to the healthy control group, the relative abundances of the stress-tolerant phenotype (*P* = 0.011) and the potentially pathogenic phenotype (*P* = 0.041) were significantly reduced in the AK group, whereas the differences in other phenotypic functions were not statistically significant. This suggests that the conjunctival sac microbiota itself may not exhibit direct pathogenicity.

To explore the potential impact of *Achromobacter* on *Acanthamoeba* adhesion and migration, we conducted an in vitro adhesion and migration assay. The adhesion assay revealed a statistically significant enhancement of *Acanthamoeba* binding in the Ac+Ach group (*Acanthamoeba* with *Achromobacter*) compared to the Ac group (*Acanthamoeba* alone), with a higher mean adhesion rate in Ac+Ach (83.33% ± 3.51% vs. 66.67% ± 1.89%, *P* = 0.007; Fig. 4B), indicating that *Achromobacter* was associated with increased *Acanthamoeba* adhesion to the corneal epithelium. Furthermore, the migration assay demonstrated that *Achromobacter* was associated with enhanced *Acanthamoeba* motility, with larger migration areas observed at 24, 48, and 72 hours in the Ac+Ach group compared to the Ac group (all *P* < 0.05; Fig. 4C).

### *Achromobacter* as a Potential Endosymbiont Influencing *Acanthamoeba* Through Metabolites

**Figure 5.**
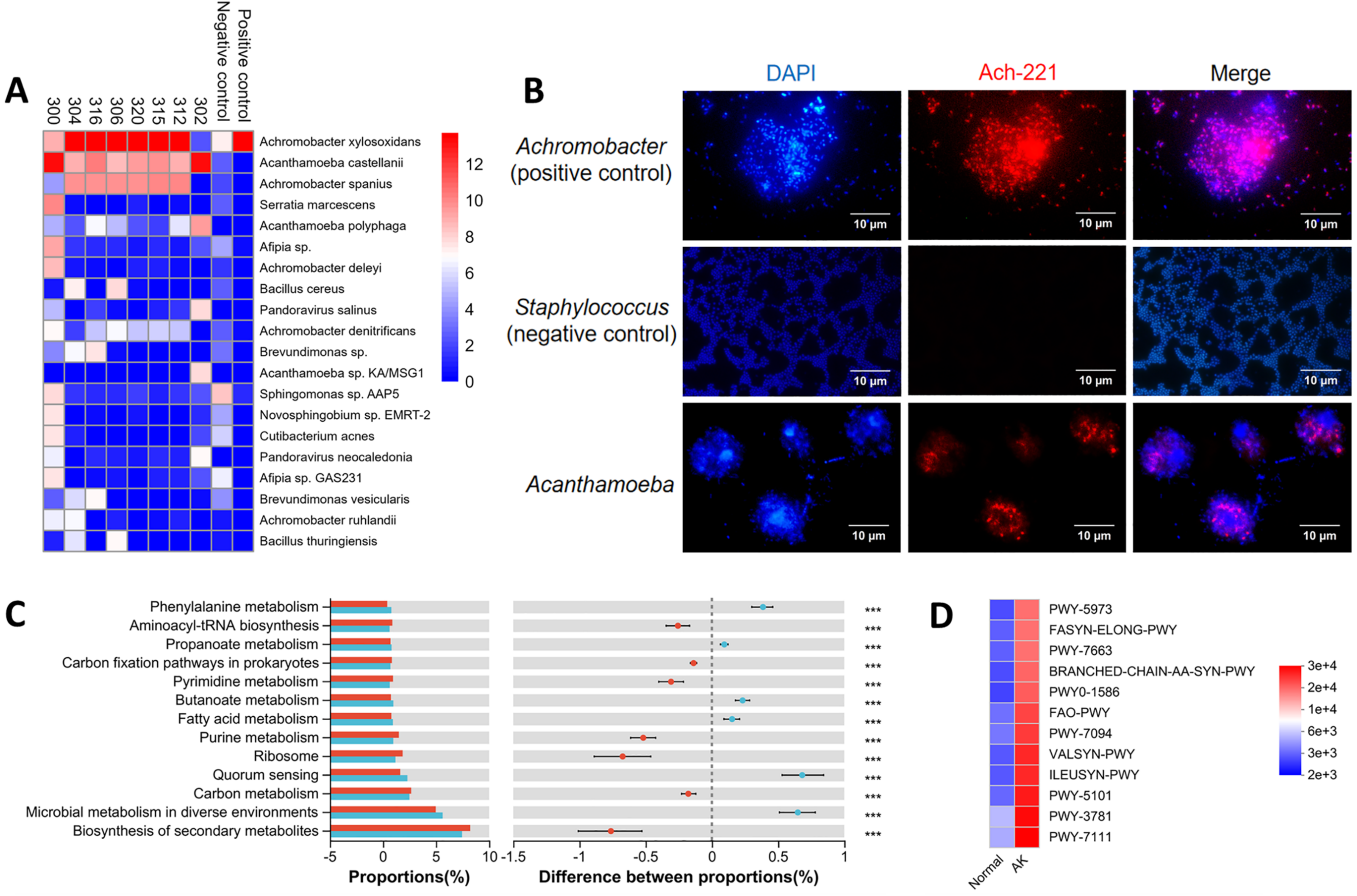
*Achromobacter* as a potential endosymbiont influencing *Acanthamoeba* through metabolites. (**A**) Taxonomic distribution of the predicted species in *Acanthamoeba* extracted from patients with AK through metagenomic analysis, along with positive and negative controls. The number of predicted species was indicated by log (NTrPM+1). (**B**) Representative FISH image showing *Achromobacter* (positive control = *red*), *Staphylococcus* (negative control), and *Achromobacter* (*red*) signal within *Acanthamoeba*. Nuclei were counterstained with DAPI (*blue*). (**C**) KEGG Pathway Level 3 with *P*_adjust < 10^−4^ was highlighted through the comparison between the AK and the healthy control groups. (**D**) Metabolic pathways identified by MetaCyc analysis, suggesting metabolic changes between the AK and the healthy control groups. *** Represents *P* < 0.001.

The metagenomic analysis results (Fig. 5A) showed that the top microbial species found within *Acanthamoeba* extracted from eight patients with AK, ranked by relative abundance, were: *Achromobacter xylosoxidans*, *Achromobacter spanius*, *Acanthamoeba castellanii*, and *Acanthamoeba polyphaga*. *Achromobacter* spp. dominated the endosymbiotic population in nearly all groups except for sample 302. These *Acanthamoeba* isolates were identified as belonging to four T4 genotypes: T4A (*n* = 4), T4B (*n* = 2), T4D (*n* = 1), and T4E (*n* = 1) (see the Table). Notably, the positive control was predominantly composed of *Achromobacter xylosoxidans* (99.9% of the total NTrPM), confirming detection specificity, whereas the negative control samples showed higher relative abundances of *Ralstonia insidiosa* (57.0% of the total NTrPM) and *Herbaspirillum huttiense* (12.5% of the total NTrPM), confirming that the detected signals are specific. FISH further confirmed the intracellular localization of *Achromobacter* within *Acanthamoeba* trophozoites, demonstrating dense bacterial clusters (red signals) colocalized with amoebae (Fig. 5B). The specificity of the FISH probes was validated using *Achromobacter* as a positive control and *Staphylococcus* as a negative control, confirming that the observed signals accurately represent *Achromobacter*. This finding suggests a potential association between *Achromobacter* spp. and *Acanthamoeba* pathogenesis through symbiotic interaction.

To further investigate whether the formation of an endosymbiotic relationship between bacteria and *Acanthamoeba* leads to specific metabolic changes in the bacteria, we conducted a PICRUSt2 analysis, which inferred the potential metabolic pathways involved. We first analyzed KEGG Pathway Level 1 (Supplementary Fig. S3A), revealing that metabolism accounted for the largest proportion, with no significant differences observed in overall metabolic levels between the two groups (the healthy control group = 75.78 ± 0.72 vs. the AK group = 76.26 ± 0.32, *P* = 0.379; Supplementary Fig. S3B). Subsequently, we examined KEGG Pathway Level 2 (Supplementary Fig. S3C), which suggested that amino acid metabolism was more enriched in the AK group compared to the healthy control group (8.78 ± 0.27 vs. 7.55 ± 0.43, *P* < 0.001), as well as lipid metabolism (2.23 ± 0.16 vs. 2.50 ± 0.05, *P* < 0.001; Supplementary Fig. S3D).

When analyzing KEGG Pathway Level 3 (Fig. 5C), we compared these pathways between the AK and the healthy control groups. Notably, core pathways, including phenylalanine metabolism, propanoate metabolism, butanoate metabolism, and fatty acid metabolism, were significantly upregulated in the AK group (all *P* < 0.001). In contrast, pathways such as purine metabolism and pyrimidine metabolism were markedly downregulated (both *P* < 0.001). Furthermore, MetaCyc analysis identified the top three pathways as pyruvate fermentation to isobutanol (PWY-7111), aerobic respiration I (PWY-3781), and L-isoleucine biosynthesis II (PWY-5101) in the AK group compared to the healthy control group (all *P* < 0.001; Fig. 5D).

## Discussion

This study systematically analyzed the changes in the conjunctival sac microbiota of patients with AK, focusing on the critical role of *Achromobacter* in AK pathogenesis. Through 16S rRNA sequencing and metabolic function prediction, we observed the enrichment of *Achromobacter* in conjunctival sac microbiota and suggested its potential association with promoting *Acanthamoeba* infection through alterations of metabolic pathways.

*Achromobacter*, a genus of non-fermenting Gram-negative bacteria, are widely distributed in moist environments and have been increasingly recognized as opportunistic pathogens in various clinical settings.^24^ These bacteria possess intrinsic resistance mechanisms, enabling them to survive under adverse conditions, including antimicrobial pressure.^25^ *Achromobacter* is known for its ability to form biofilms, secrete extracellular enzymes, and adapt to host environments, all of which contribute to its pathogenic potential.^25^^,^^26^ In recent years, its role in altering host microbial communities and promoting disease progression has garnered significant attention.^26^^,^^27^ Among conjunctival sac microbiota of patients with AK, *Achromobacter* demonstrated a significant increase in abundance, dominating the microbial community and standing in stark contrast to healthy controls. Notably, *Achromobacter* abundance was also elevated in the unaffected eyes of patients with AK compared with healthy controls, whereas no significant differences in *Achromobacter* abundance were observed between the affected and unaffected groups. This suggests that its enrichment is not solely due to endosymbiosis with *Acanthamoeba*, but may reflect a broader microbial imbalance associated with a disease-prone ocular microenvironment. These changes may weaken the ecological balance of the conjunctival sac microbiota, impair its defensive functions, and create favorable conditions for *Acanthamoeba* colonization and proliferation, indicating that *Achromobacter* may play an important role of AK.

Previous studies have shown that certain endosymbionts (e.g. *Pseudomonas*, *Legionella*, *Mycobacteria*, *Aspergillus*, and *Escherichia*) can enhance protozoan pathogenicity by providing nutritional support or protecting hosts from environmental stress.^28^^–^^33^ Our study identified *Achromobacter* as the most abundant endosymbiont within *Acanthamoeba*, consistent with the 16S rRNA sequencing results. Interestingly, this aligns with recent findings showing that *Acanthamoeba* isolated from the cornea harbor significantly higher abundances of *Achromobacter* compared with environmental water sources.^34^ In addition, we demonstrated that *Achromobacter* boosted *Acanthamoeba* adhesion and migration. This finding suggests that *Achromobacter* may indirectly support *Acanthamoeba* invasion through endosymbiosis, highlighting its potential role in enhancing the pathogenicity of *Acanthamoeba.*^24^^,^^27^ Previous studies have demonstrated that *Acanthamoeba* isolates containing multiple bacterial endosymbionts display significantly enhanced cytopathic effects in vitro, suggesting that these symbionts may contribute to increased protozoan virulence.^31^ Moreover, *Pseudomonas aeruginosa* has been shown to synergize with *Acanthamoeba* in vivo, aggravating the severity of corneal infections.^35^ Recent clinical evidence also suggests that endosymbiont-positive AK cases are associated with more severe corneal infiltrates and an increased need for surgical interventions, including therapeutic keratoplasty.^36^ These findings underscore the dual role of bacterial endosymbionts in both promoting protozoan virulence and influencing clinical outcomes. Functional predictions further revealed that *Achromobacter* may be associated with *Acanthamoeba* infection through metabolic regulation. *Achromobacter* may impact the local microenvironment through the secretion of various metabolites, thereby indirectly supporting the growth and pathogenicity of *Acanthamoeba.*^37^ Studies indicate that *Achromobacter* secretes long-chain fatty acids, such as palmitic acid and oleic acid, which are key players in the activation of the fatty acid metabolism pathway.^25^^,^^37^ These metabolites serve as energy-rich substrates associated with *Acanthamoeba*’s motility and provide structural components for its membrane synthesis, ensuring cellular integrity and promoting proliferation.^38^

Additionally, *Achromobacter* may enhance the amino acid metabolism pathway by releasing amino acids like glutamate and proline.^37^^,^^39^^,^^40^ These metabolites may be essential precursors for protein synthesis, fulfilling *Acanthamoeba*’s metabolic demands and supporting its rapid growth. Proline, in particular, plays a critical role in osmoregulation, enabling *Acanthamoeba* to thrive in hyperosmotic environments.^40^ In the propanoate metabolism pathway, *Achromobacter* produces short-chain fatty acids (SCFAs), such as propionate and butyrate.^37^ These SCFAs may not serve as direct energy sources for *Acanthamoeba* and modulate the local pH, weakening the host's immune defenses and facilitating *Acanthamoeba* colonization and invasion.^41^^,^^42^ Concurrently, *Achromobacter*-driven aerobic respiration I efficiently generates ATP through oxidative phosphorylation, meeting high energy demands for motility and phagocytosis of *Acanthamoeba.*^37^ Additionally, the byproducts of propanoate metabolism may influence host inflammatory responses, further contributing to an environment conducive to *Acanthamoeba* presence.^43^

From a clinical perspective, the significant enrichment of *Achromobacter* provides new insights for AK diagnosis and treatment. On the one hand, *Achromobacter* could serve as a potential biomarker for the early identification of susceptible patients. On the other hand, targeted interventions aimed at disrupting key metabolic pathways involved in *Achromobacter-Acanthamoeba* interactions may offer novel therapeutic strategies. Furthermore, approaches to restore the balance of the conjunctival sac microbiota, such as probiotics or selective antimicrobial agents, may also improve outcomes in patients with AK.

Although this study offers valuable insights, several limitations must be acknowledged. First, the relatively small sample size limits the generalizability of the findings, and larger studies are needed to validate these observations. Second, in the control group, only right eye samples were collected, which may raise concerns about sampling bias. Third, although this study offers valuable insights, it cannot establish a causal relationship between *Achromobacter* enrichment and AK. The cross-sectional design limits the ability to determine whether *Achromobacter* contributes to the development of AK or is a consequence of the disease. Future longitudinal and mechanistic studies are needed to clarify this association and elucidate the dynamic changes in *Achromobacter* during disease progression. Last, whereas functional predictions provide critical clues, further in vitro and in vivo experiments are necessary to confirm the metabolic interactions between *Achromobacter* and *Acanthamoeba*.

In conclusion, this study highlights a potential role of *Achromobacter* in AK, offering new perspectives on the relationship between microbiota dysbiosis and AK pathogenesis. These findings lay a theoretical foundation for *Achromobacter*-based diagnostic and therapeutic strategies and provide new directions for improving AK management. Further research on the interactions between *Achromobacter* and *Acanthamoeba* will be instrumental in developing precise interventions for AK treatment.

## Supplementary Material

Supplement 1

Supplement 2
