# Supplementary material for: Achromobacter in the Conjunctival Sac Microbiota: Potential Association With Acanthamoeba Keratitis Related to Orthokeratology Lenses
Source: Invest Ophthalmol Vis Sci. 2025 Jul 30;66(9):71. doi: 10.1167/iovs.66.9.71 (PMC12315931; doi:10.1167/iovs.66.9.71)
Supplement: Supplement 1 [file iovs-66-9-71_s001.pdf]

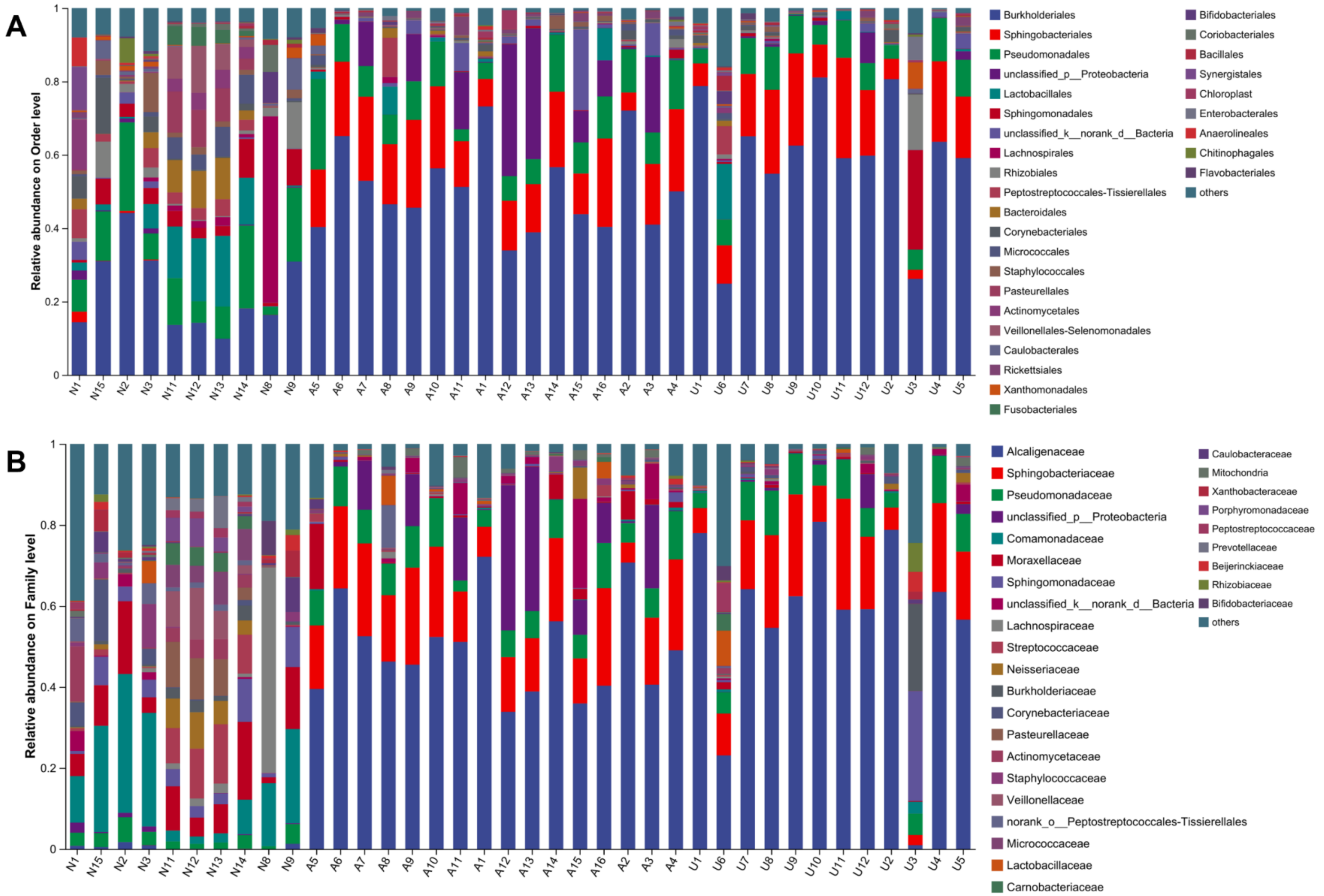

Figure S1. Barplot showing the relative abundances of bacterial (A) orders and (B) families in the normal and AK groups.

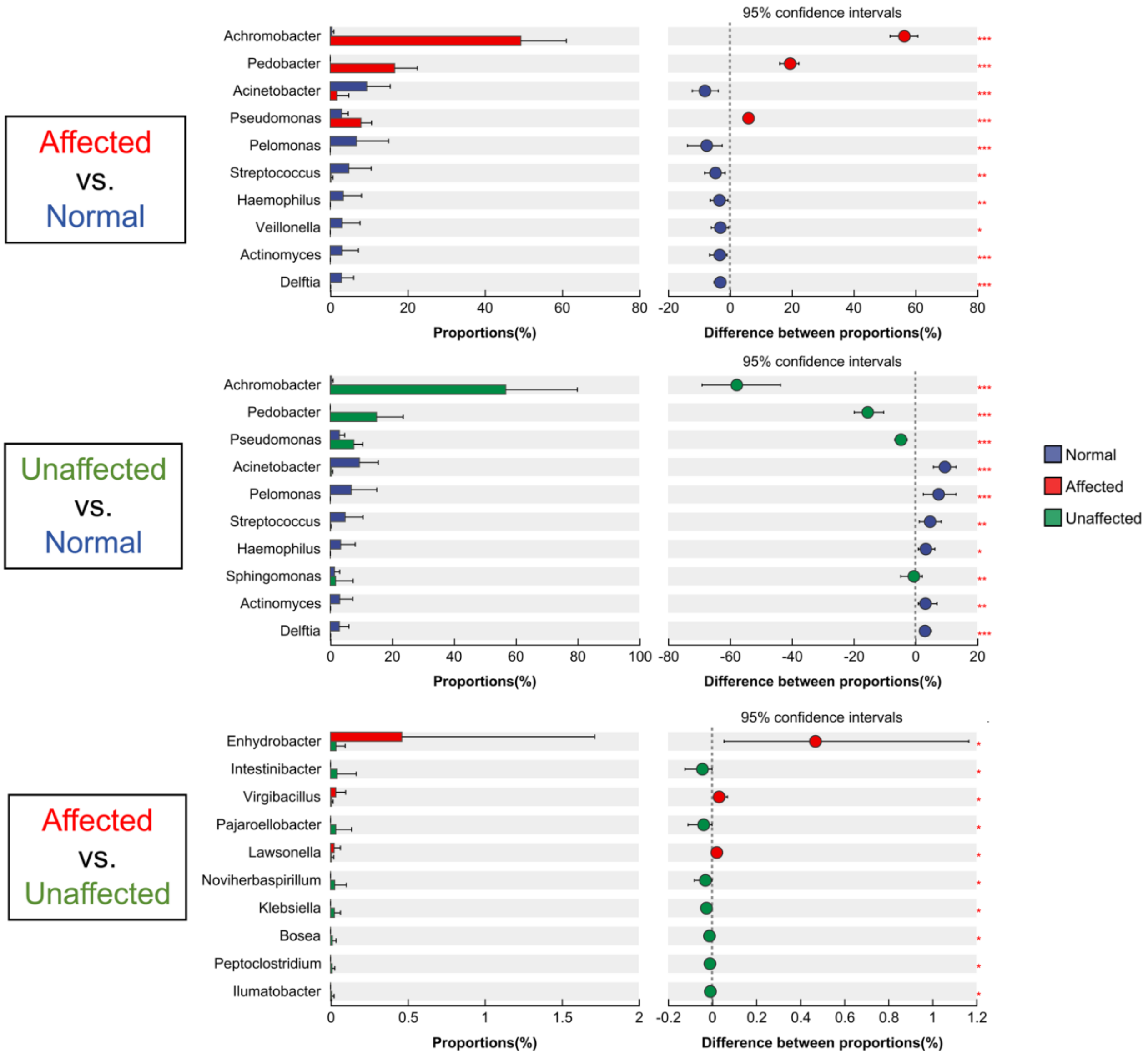

Figure S2. Comparison of the relative abundance of key genera among affected group, unaffected group, and normal group.

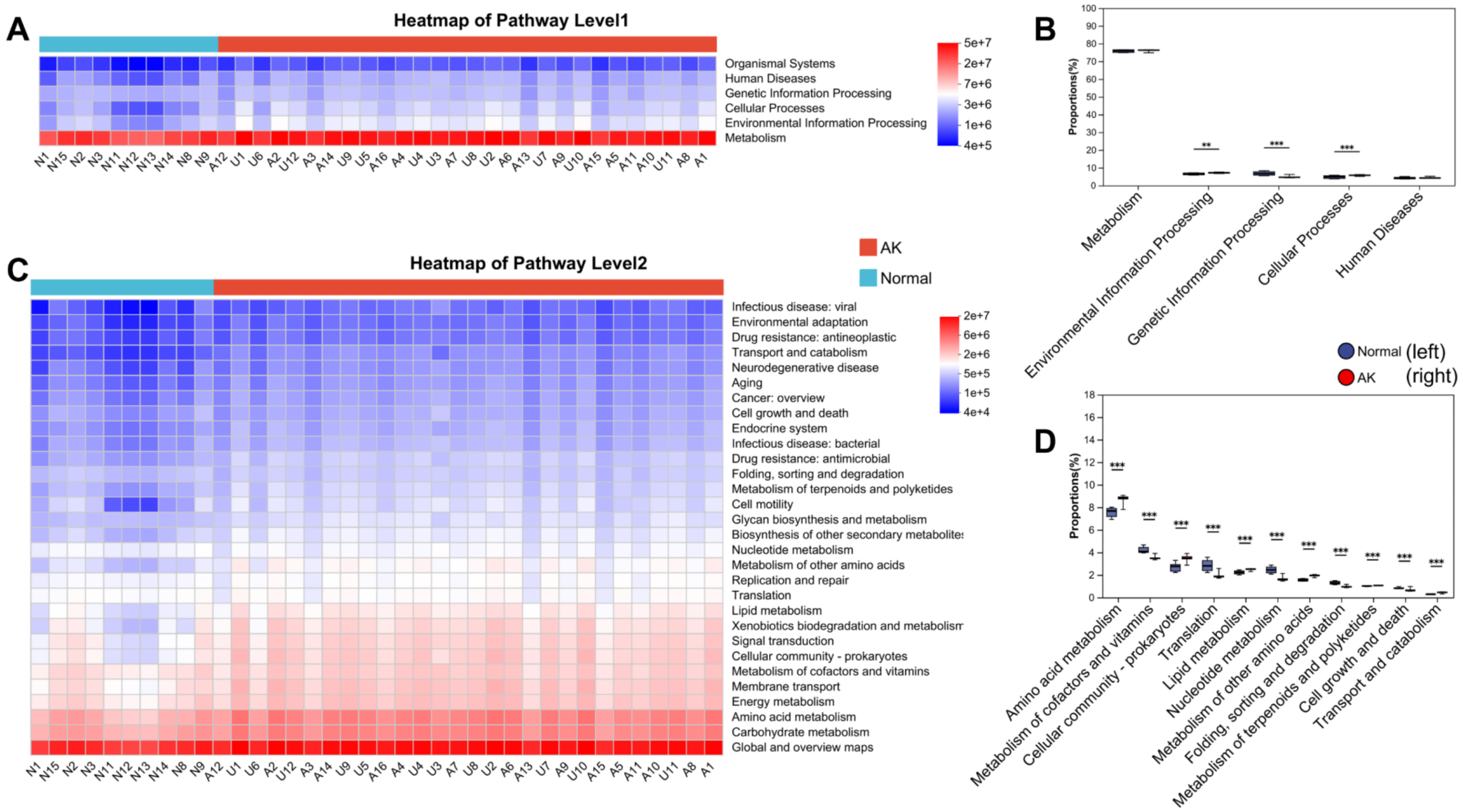

Figure S3. KEGG Pathway Level 1 and KEGG Pathway Level 2 analysis of bacterial metabolic changes. Heatmap of (A) KEGG Pathway Level 1 and (C) KEGG Pathway Level 2 between AK and normal groups, with the number representing functional abundance. Pathways of (B) Level 1 and (D) Level 2 highlight through the comparison between the AK and normal groups. ** represents $p < 0.001$. *** represents $p < 0.001$.
