# Supplementary material for: Achromobacter in the Conjunctival Sac Microbiota: Potential Association With Acanthamoeba Keratitis Related to Orthokeratology Lenses
Source: Invest Ophthalmol Vis Sci. 2025 Jul 30;66(9):71. doi: 10.1167/iovs.66.9.71 (PMC12315931; doi:10.1167/iovs.66.9.71)
Supplement: Supplement 2 [file iovs-66-9-71_s002.pdf]

Supplementary Material

Table S1. Sequencing information statistics

Sample_name	Raw sequence information	Denoised sequence information		Effective sequence information	
	Seq_num	ASVs_num	Seq_num	ASVs_num	Seq_num
U1	70779	359	61536	291	12373
A1	63793	315	57353	292	12373
U2	62110	156	56284	128	12373
A2	58276	149	52030	141	12373
U3	221358	1418	148792	726	12373
A3	78405	144	69438	126	12373
U4	73154	72	62782	61	12373
A4	77141	253	64734	238	12373
U5	65892	212	56195	188	12373
A5	78057	163	65137	149	12373
A6	76362	83	64332	74	12373
A7	77723	103	67022	89	12373
U6	78364	1111	65520	934	12373
A8	89905	192	75468	169	12373
U7	70754	147	59913	131	12373
A9	66997	127	58318	101	12373
A10	70874	199	59779	190	12373
U8	77430	166	65686	150	12373
A11	68017	298	60145	222	12373
U9	78021	57	65480	52	12373
A12	92809	145	83002	119	12373
U10	73955	101	62673	88	12373
A13	92442	157	82723	129	12373
A14	77698	117	65431	109	12373
A15	67794	219	60242	183	12373
U11	73776	38	62269	35	12373
A16	73239	111	62638	102	12373
U12	74884	104	63945	95	12373
N1	58521	536	53121	454	12373
N2	144281	2047	107335	1036	12373
N3	118746	1659	82209	1081	12373
N4	54231	339	51247	316	12373
N5	41807	176	38332	173	12373
N6	40171	226	29488	219	12373
N7	44844	224	32243	216	12373
N8	42647	209	31383	203	12373
N9	45097	258	33628	254	12373
N10	41200	206	38093	206	12373
